# Theory and conception of Somatopsyche Psychiatric Intervention: a transdiagnostic body-mind intervention for psychiatric practice

**DOI:** 10.3389/fpsyt.2025.1644739

**Published:** 2025-08-19

**Authors:** Renata Fáro Guerra, Hermano Tavares

**Affiliations:** ^1^ Outpatient Program of Habit and Lifestyle Change at the Institute of Psychiatry, University of Sao Paulo (USP), São Paulo, Brazil; ^2^ Psychiatry Department, School of Medicine at USP, São Paulo, Brazil

**Keywords:** body awareness, body-mind therapies, interoception, meditation, psychiatry

## Abstract

This article introduces the Somatopsyche Psychiatric Intervention (SPI), a novel body-mind approach that integrates body awareness practices, meditation, and contemporary neuroscience theories in the treatment of psychiatric conditions. SPI is structured in seven steps across eight weekly sessions, aiming to enhance emotional regulation, decision-making, and patient resilience. While the model is still undergoing empirical validation and should be considered primarily theoretical at this stage, it has been implemented biannually in a clinical outpatient setting since 2019. Preliminary data collection using validated instruments (e.g., the MAIA scale) is ongoing and will inform future publications. The SPI represents a promising integrative framework in mental health care, although challenges remain regarding the training of professionals and adaptation by patients used to more traditional methods. Further studies, including pilot trials and comparative analyses, are planned to rigorously evaluate its effectiveness.

## 1 Introduction

Contemporary psychiatry has achieved remarkable advancements in understanding and treating mental disorders, with significant progress in evidence-based pharmacological and psychotherapeutic approaches. However, despite these advancements, the field still faces the challenge of holistically integrating the body-mind dimension into routine clinical practice. Often, the emphasis falls on symptom modulation through predominantly “top-down” (from thought to body) interventions, which, while effective, may neglect the profound and bidirectional interconnection between somatic states and subjective psychic experience—a theme that has been revisited since historical discussions on body-mind dualism (1). The term “somatopsyche” itself has roots tracing back to early 20th-century medical literature, reflecting a long-standing recognition of the body’s primacy in subjective experience (2). This gap becomes even more evident in the absence of structured and protocolized body-mind interventions that systematically address somatic dynamics in the genesis and maintenance of mental health.

It is in this context that the Somatopsyche Psychiatric Intervention (SPI) emerges as an innovative proposal. Our work aims to fill this fundamental gap by introducing a manualized, clinically promising, and theoretically grounded therapy model designed to integrate contemplative practices, body awareness, and recent advances in neuroscience into psychiatric clinical practice. SPI distinguishes itself by its primary emphasis on interoception – the perception of internal bodily sensations – an increasingly recognized cornerstone crucial for emotional regulation, enhanced decision-making, and the construction of a robust sense of an *embodied self* (3–5).

The choice of interoception as SPI’s central mechanism is not arbitrary. The growing body of neuroscientific evidence demonstrates that interoceptive awareness is intrinsically linked to self-regulation capacity, the experience of present moment awareness, and the development of personal agency (3, 6). Interoceptive dysfunctions, conversely, are frequently associated with a wide range of psychopathology, from anxiety and depressive disorders to difficulties in impulse regulation and trauma recovery (7–11). This centrality of interoception positions SPI as an inherently transdiagnostic intervention, meaning it can act on underlying mechanisms shared across various disorders, aligning with the proposals of contemporary frameworks such as the Research Domain Criteria (RDoC) or the Hierarchical Taxonomy of Psychopathology (HiTOP). This transdiagnostic perspective is supported by approaches targeting common mechanisms across mental disorders (12, 13). By focusing on core processes of human experience, SPI transcends traditional diagnostic boundaries, offering a promising path for patients with diverse clinical presentations.

Beyond symptom reduction, SPI aims to foster patient well-being, personal development, and resilience, as well as to optimize emotional regulation and decision-making. This broader vision of mental health resonates with models emphasizing the central importance of positive psychology and recovery-oriented approaches in clinical practice, as advocated by Bohlmeijer and Westerhof (14) and Slade (15). By promoting a deeper integration between body and mind, SPI aims not only to alleviate suffering but also to empower individuals towards a more fulfilling life with a greater sense of purpose.

The present article introduces the Theory and Conception of the Somatopsyche Psychiatric Intervention, detailing its conceptual framework and outlining the seven protocol steps, which aim to cultivate body awareness and promote somatic self-regulation. We thus seek to offer a new perspective for psychiatric practice, founded on the inseparability between bodily states and mental health.

## 2 Methodology of the narrative review

We conducted a narrative review to support the theoretical foundation and conceptualization of the Somatopsyche Psychiatric Intervention (SPI). This approach was appropriate for integrating perspectives from neuroscience, contemplative sciences, and clinical psychology related to the mind-body connection in psychiatry. The review aimed to identify key concepts and mechanisms that inform the seven-step structure of SPI.

This methodology was suitable for synthesizing constructs from diverse conceptual domains — including contemplative practices, body psychotherapy, and transdiagnostic models in psychiatry. It allowed for the integration of emerging hypotheses and established findings, supporting the construction of a cohesive framework for a novel clinical intervention in its early developmental stage.

### 2.1 Search strategy and information sources

A broad and iterative search strategy was employed across major electronic databases, including PubMed/MEDLINE, PsycINFO, and Web of Science. The search was conducted from database inception up to July 2025 using a combination of keywords related to the core theoretical pillars of SPI. These keywords, applied in various combinations, included but were not limited to: “body-mind intervention”, “body awareness”, “interoception”, “proprioception”, “autonomic regulation”, “polyvagal theory”, “somatic marker hypothesis”, “mindfulness”, “contemplative practices”, “psychiatry”, “mental health”, “transdiagnostic approach”, “emotional regulation”, “embodiment”, “neuroscience of emotions”, “psychiatric comorbidities”.

### 2.2 Selection process and data synthesis

The initial search yielded a large number of articles. Titles and abstracts were screened for relevance to the body-mind connection, psychiatric applications, and theoretical models related to body awareness and emotional regulation. Full-text articles were then retrieved and reviewed for in-depth analysis. The selection was guided by the need to include foundational theoretical texts, key empirical studies, and comprehensive review articles that elucidated the mechanisms of action and clinical implications of body-mind practices in mental health. Special attention was given to literature that explored the transdiagnostic potential of body-based interventions and emerging neurobiological correlates.

The selected literature was then thematically synthesized. This involved identifying recurrent concepts, converging evidence, and critical gaps in the understanding of how bodily processes influence mental states and vice versa. The authors, drawing on their clinical and academic expertise in psychiatry and body-mind therapies, integrated these findings to construct the conceptual model of SPI, detailing its components, underlying mechanisms, and proposed therapeutic benefits. This iterative process of literature review and conceptual mapping allowed for the systematic articulation of SPI as a structured and evidence-informed intervention.

## 3 Theoretical background of Somatopsyche Psychiatric Intervention

The Somatopsyche Psychiatric Intervention (SPI) is grounded in a rich theoretical lineage that integrates historical perspectives on the body-mind connection with contemporary neuroscience and contemplative science. This section elucidates the foundational concepts that underpin SPI, establishing its scientific rationale and unique contributions.

While the literature reviewed provides robust support for each individual mechanism incorporated into SPI — such as interoception, autonomic regulation, and contemplative practices — it is important to note that few studies to date have tested integrative protocols that combine these components within psychiatric care. Moreover, many of the empirical findings cited stem from studies of isolated practices (e.g., mindfulness meditation or interoceptive training) in general populations rather than clinically diagnosed individuals. This underscores both the innovative potential and the current limitation of SPI: although it is built upon well-established mechanisms, the synergistic effect of their integration within a structured psychiatric intervention remains hypothetical. Future empirical studies must evaluate whether this integration enhances outcomes compared to component interventions. Therefore, our review is intentionally interpretative and conceptual, aiming to articulate a new framework rather than to establish empirical efficacy at this stage.

### 3.1 Historical and conceptual foundations of the body-mind relationship

The intricate relationship between the body and mind has been a subject of philosophical and scientific inquiry since antiquity. Early perspectives, such as those of Hippocrates (460–370 BC), viewed the human being as a functional unity, where the psyche played a regulatory role over the body. This holistic view was notably challenged in the 17th century by René Descartes, who established a dualistic concept, treating the body as a machine separate from the mind. Despite its influence, this dualism inadvertently fostered a fragmentation in understanding human health, often leading to a reductionist focus on either mental or physical phenomena (1).

However, counter-currents to Cartesian dualism emerged, re-emphasizing the profound interaction between psychic and somatic states. Johann Heiroth introduced the term “psychosomatic” in 1818, and later, coined “somatopsyche” in 1828, both suggesting that the mind could influence the body and vice versa (1). The term “somatopsyche” specifically emphasizes the primacy of the body in constituting subjective experience, suggesting that mental phenomena emerge from bodily processes. Recent historical reviews further trace the use of “somatopsyche” back to early 20th-century medical literature, providing a broader historical grounding for this conceptual inversion (2). This enduring fascination with the body’s role in mental life continued, influencing figures like Sigmund Freud (1856–1939), who, while focusing on mental intervention, recognized that “the ego is first and foremost a bodily ego” (16). Later, pioneers in body-oriented therapeutic approaches, such as Wilhelm Reich (1897–1957) and Alexander Lowen, highlighted the inseparable connection between mind and body, introducing concepts like “muscular armor” and developing methods centered on bodily expression and release (17, 18).

A pivotal shift in the scientific understanding of the body-mind relationship occurred with the work of neurologist Antonio Damasio. His seminal book, “Descartes’ Error” (19), revolutionized the field by exploring the Somatic Marker Hypothesis. Based on studies of neurological patients with prefrontal cortex injuries, Damasio demonstrated that individuals unable to learn from emotional mistakes exhibited impaired decision-making, even with preserved cognition. He posited that “somatic markers,” represented by physical sensations associated with emotional states, serve as crucial signals that assess situations and guide behavior (19). The absence of these markers leads to less effective decisions, highlighting the fundamental role of emotions and bodily sensations in reasoning and decision-making processes (20).

This theory has profound implications for understanding various psychiatric disorders involving emotional dysregulation, impulse control, and empathy deficits (9, 21, 22). Unlike traditional psychiatry, which often emphasizes top-down cognitive approaches, this perspective, akin to the historical “somatopsyche” concept, posits a bidirectional model where interoception, proprioception, and autonomic regulation are central to understanding mental health.

This bidirectional understanding of body-mind interaction also underpins contemplative practices such as mindfulness, which have demonstrated clinical benefits through their influence on interoception, emotional regulation, and attentional processes. Mindfulness practices may exert their therapeutic effects through mechanisms including enhanced interoception, attentional control, and emotion regulation (23), all of which align closely with the therapeutic targets of SPI.

### 3.2 Body awareness and embodiment

The growing interest in neuroscience and its correlation with emotions has significantly illuminated the intricate relationship between the body and mind. Central to this understanding is the construct of body awareness, defined as a sense of body ownership and agency (3). This multidimensional concept encompasses three primary dimensions: proprioception, interoception, and mindfulness (4).

- Proprioception refers to the spatial awareness of the body, including its position, orientation, and the perception of articular and muscular tension (4). It provides a continuous stream of information about the body’s physical state in space.
- Interoception is a multidimensional construct that describes how individuals receive, evaluate, and respond to their internal bodily sensations. While proprioception focuses on the musculoskeletal system, interoception is akin to visceral cognizance—an awareness of one’s own physiological state (24). The insular cortex, responsible for integrating interoceptive and exteroceptive signals, plays a pivotal role in generating subjective feelings (4, 11, 22). Interoception is critical for affect regulation, decision-making, and the sense of an “embodied self,” which is the feeling of being present in one’s own body with integrated perceptions, thoughts, and emotions (5). The perception of body sensations can be either useful, providing a sense of motivation, embodiment, and well-being, or catastrophic, as seen in panic and anxiety disorders (3, 7).
- *Mindfulness, usually achieved through meditation and contemplative practices, is a mental state attained by directing attention to the present moment without judgment towards one’s feelings, thoughts, and bodily sensations* (25). It has been shown to increase positive affect, reduce negative affect, and promote psychological well-being, contributing to neuroplastic changes in areas like the prefrontal cortex and insula (26, 27). Numerous meta-analyses have confirmed the effectiveness of mindfulness is beneficial for chronic pain, stress, and many physical and mental conditions (28). Goldberg et al. (29) found that mindfulness-based interventions show moderate to strong effects across psychiatric conditions, supporting the contemplative elements integrated in SPI. For instance, Eberth and Sedlmeier (30) demonstrated significant reductions in anxiety, depression, and stress-related symptoms across diverse populations.

As individuals deepen their attunement to interoceptive and exteroceptive signals, body awareness evolves, fostering complementary senses of presence and agency (3). Presence, or connection with the present moment, enhances well-being, particularly in meditative practice, while agency promotes purposeful action and self-regulation (3). This evolution is further supported by neurobiological mechanisms that regulate emotional states. In this context, recent research on the glymphatic system, responsible for the brain’s cleansing and regulation of neurobiological functions during sleep and rest, suggests that it may play a crucial role in mental health. The proper functioning of the glymphatic system may be linked to the brain’s capacity to process and regulate emotions, which is vital for maintaining emotional well-being. Incorporating practices that enhance the regulation of this system, such as those promoted in SPI, could represent a new frontier for understanding and treating psychiatric conditions (31).

The process that patients experience through body-mind therapies can be understood as a progression towards a deeper integration of body and self, termed levels of embodiment. This maturation can be conceptualized in stages (32):

1. “The absent body”: The body is taken for granted, often described as distant.
2. “The objectified bodily state”: The body is experienced in opposition to the “self,” often symptomatic (e.g., pain, loss of function).
3. “Cultivated Immediacy Relationships”: A new, accepting relationship with the body, lived without objectification.
4. “The subjective body”: The body is experienced as a source of meaning, learning, and intelligence, gaining a new perspective as a locus of consciousness.

Building upon these established levels, the Somatopsyche Psychiatric Intervention proposes a unique fifth level: “Self-watcher.” This concept, born from the integration of body-mind theory and contemplative practice, signifies an advanced stage where the individual’s body awareness deepens to a point of being able to observe bodily sensations, emotions, and thoughts as a spectator, without being overwhelmed or confused by symptoms. This allows for a metacognitive detachment that promotes insight and emotional regulation, fostering a more resilient and integrated sense of self.

This advanced level of embodiment, characterized by metacognitive awareness and emotional equanimity, also finds support in recent neurobiological findings. Garland et al. (33) highlighted that mindfulness interventions influence biobehavioral pathways, including vagal tone and reward circuits, providing neurobiological support for interventions like SPI.

### 3.3 Polyvagal theory and neuroception

The Polyvagal Theory, developed by Stephen Porges (34), provides a neurophysiological framework that enriches the body-mind model of SPI, particularly in relation to emotional regulation and perceived safety. Rather than elaborating on the entire evolutionary trajectory of the autonomic nervous system, we emphasize the clinically relevant insight that emotional and behavioral responses to stress are hierarchically organized and mediated by distinct branches of the vagus nerve.

Central to SPI is the idea that interoceptive and proprioceptive cues can influence autonomic tone and shift individuals between defensive and socially engaged states. This concept derives from the polyvagal notion of neuroception — the unconscious detection of safety or threat — which regulates whether the ventral vagal complex (associated with calm and social engagement) or more primitive responses (fight, flight, freeze) are activated.

By incorporating body-based practices that promote relaxation, diaphragmatic breathing, and safe social engagement, SPI aims to facilitate the activation of the ventral vagus and foster neurophysiological states conducive to emotional regulation, insight, and connection. In this sense, Polyvagal Theory supports the neurobiological plausibility of SPI, particularly in its early steps focused on grounding and body mapping.

The theory also offers a useful explanatory model for understanding why patients under chronic stress or trauma may default to rigid autonomic states. SPI’s structured progression, which integrates awareness, agency, and interoception, is designed to gently restore flexibility in these neural circuits and promote adaptive self-regulation.

**Figure 1:**
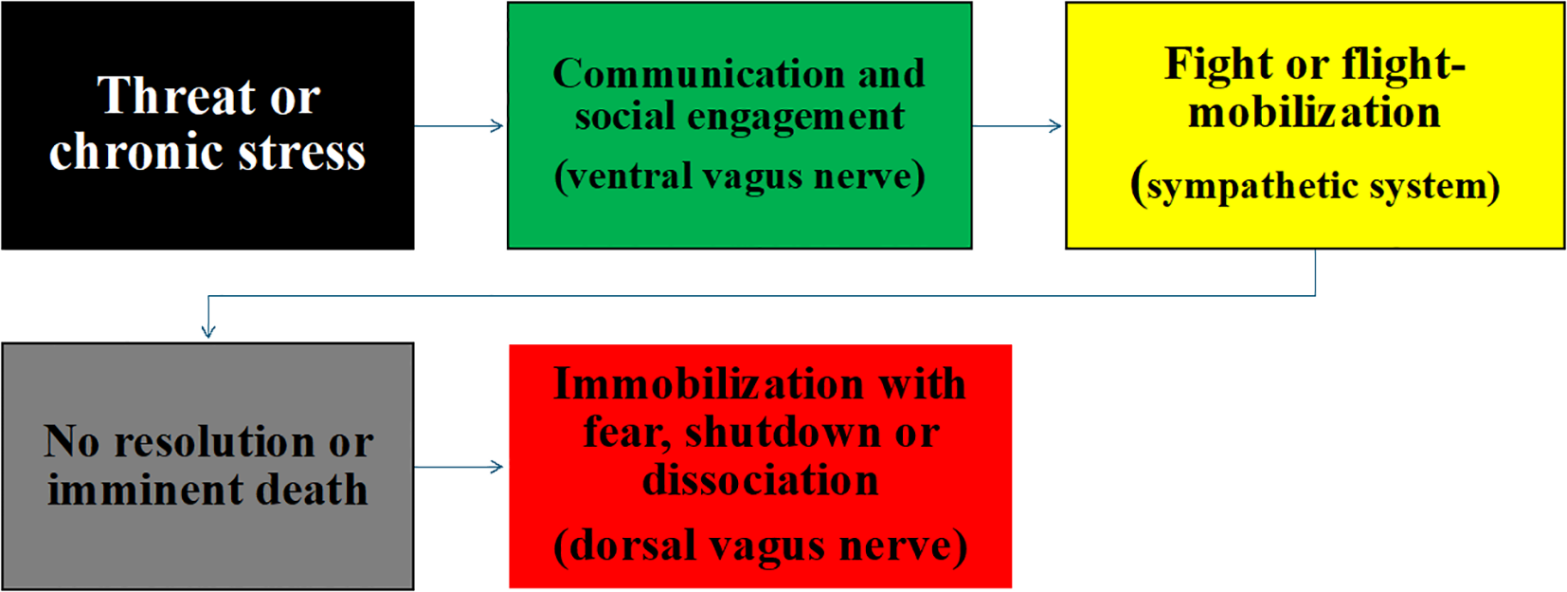
Default hierarchy of defense responses of ANS under stress. ANS, autonomic nervous system.

A fundamental principle of the Polyvagal Theory, echoing Hughlings Jackson’s neurology of the late 19th century, is that under stress, the brain returns to more primitive levels of functioning (35). Humans, when subjected to continuous threat or stress, preferentially react through social behavior; if this is not possible, they adopt defensive strategies in a regressive order of behavioral phylogeny: fight, flight, and freeze. This is often referred to as the “default hierarchy of defensive systems” of the Central Nervous System (**Figure 1**).

An intriguing aspect of this dynamic is that it can also be derived in a counterproductive manner, wherein the perception of a muscular and visceral altered state can elicit a sense of threat, whereas the relaxation of muscles and the stomach with proper tone can signal safety even in a discordant environment. This clinical perspective is echoed in Dana’s (36) work, which applies the Polyvagal Theory to psychotherapy and emphasizes the therapeutic relevance of cultivating body awareness and autonomic regulation for emotional safety and connection. The SPI directly leverages these polyvagal principles by guiding individuals through practices that enhance interoceptive awareness and promote the activation of the ventral vagal system, thereby fostering states of safety, connection, and ultimately, self-regulation.

### 3.4 The transdiagnostic imperative in psychiatry

The landscape of mental health treatment is increasingly recognizing the limitations of purely diagnostic-specific approaches. The concept of transdiagnostic treatments has gained significant traction, referring to interventions that target common underlying mechanisms across various mental disorders rather than focusing on symptoms specific to a single diagnosis (13). These approaches can be categorized into models applicable across diverse mental health challenges, modular treatments adaptable to specific patient needs regardless of diagnosis, or interventions targeting shared etiological or maintenance mechanisms for groups of disorders (12). The Somatopsyche Psychiatric Intervention (SPI) primarily aligns with this last definition, addressing core processes such as emotional dysregulation and interoceptive dysfunction that underpin a wide spectrum of psychological distress.

The transdiagnostic perspective of SPI is particularly relevant in the current global context. Emerging psychiatric literature highlights the somato-emotional impact of the COVID-19 pandemic, particularly in exacerbating dissociation, alexithymia, and suicidality in many populations. Recent studies underscore that social isolation, job loss, and constant stress are significant factors increasing the risk of these conditions (37). In this context, SPI’s focus on body-awareness practices and emotional regulation techniques becomes essential for recovery, providing patients with tools to reconnect their emotional experiences with bodily sensations and reduce emotional disconnection (38). While SPI is not recommended for acute psychotic or manic conditions, it may offer substantial benefits to subacute or post-crisis patients experiencing emotional disconnection.

Furthermore, the transdiagnostic potential of SPI extends to populations where body-mind integration is often challenged. Patients with neurodevelopmental conditions such as Autism Spectrum Disorder (ASD), who frequently experience interoceptive dysfunction and altered embodiment, may significantly benefit from adapted SPI protocols (39). Although SPI primarily targets emotional disorders, it is crucial to consider how psychiatric comorbidities, such as suicide, dissociation, or alexithymia, may influence a patient’s ability to fully engage with body-mind practices. Patients presenting with dissociative symptoms or difficulties in identifying and expressing emotions may require an adapted protocol, characterized by a gradual approach and more intensive follow-up. The intervention’s effectiveness can also be enhanced by adjustments that specifically address the impact of comorbid conditions on interoceptive awareness. These adaptations are vital for improving engagement in SPI, particularly for individuals with severe emotional dysregulation or trauma histories (8, 38). The core assertion of SPI and other body-mind therapies is that mental health relies heavily on the integration of body and mind, fostering an improved sense of embodiment as individuals engage with these practices.

## 4 The Somatopsyche Intervention: structure, principles, and target population

**Figure 2:**
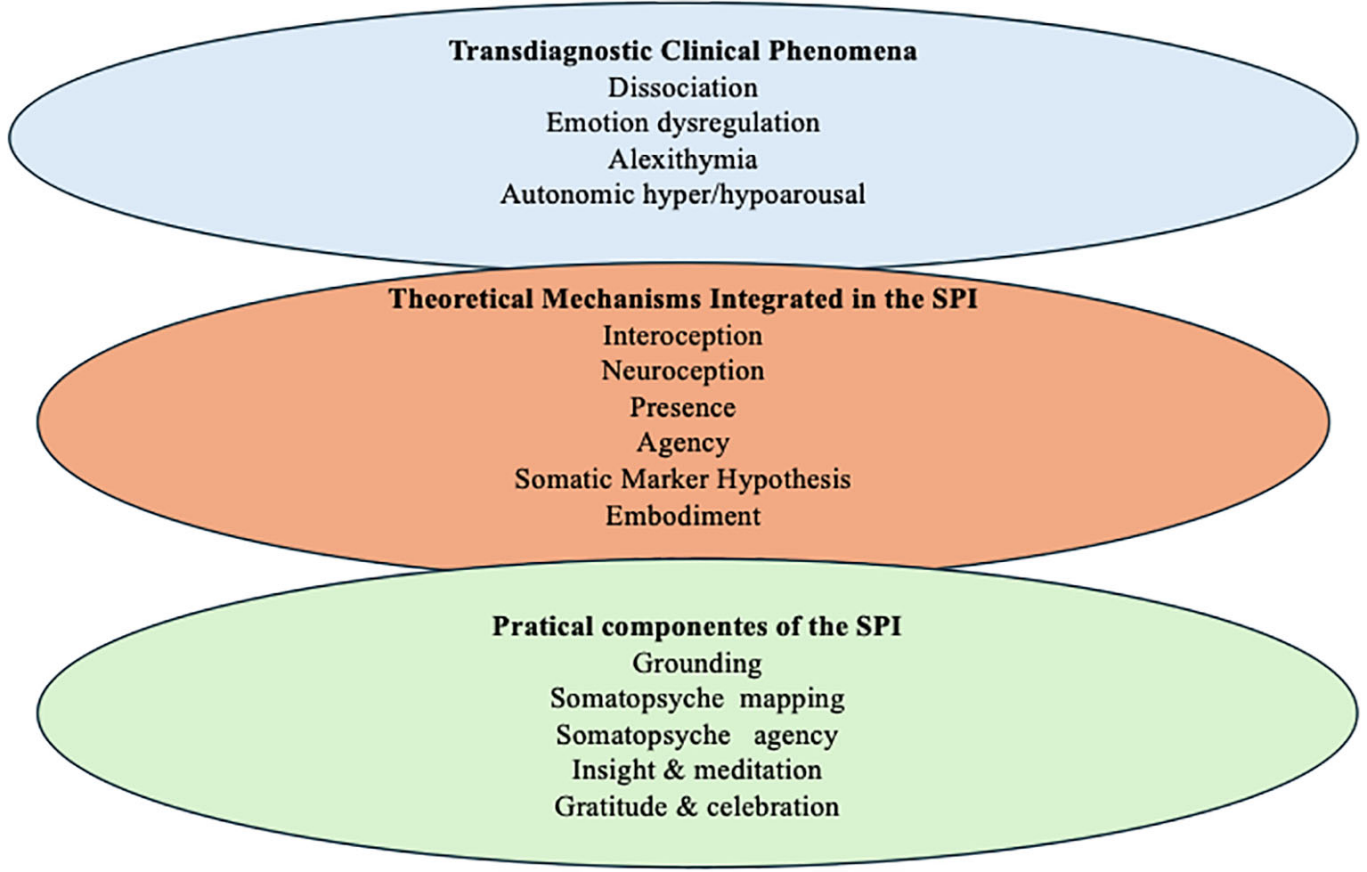
Conceptual integration of the Somatopsyche Psychiatric Intervention (SPI).

As illustrated in **Figure 2**, SPI operationalizes the connection between transdiagnostic symptoms, key body-mind mechanisms, and structured therapeutic practices. This integration provides the foundation for a unified clinical approach. The Somatopsyche Psychiatric Intervention (SPI) is an innovative and structured therapeutic approach designed to integrate contemplative practices, body awareness, and advances in neuroscience into psychiatric care.

This diagram illustrates how SPI links overlapping clinical symptoms. key transdiagnostic theoretical mechanisms, and practical intervention steps. It emphasizes the therapeutic logic from neurobiological dysfunction to embodied intervention.

**Figure 3:**
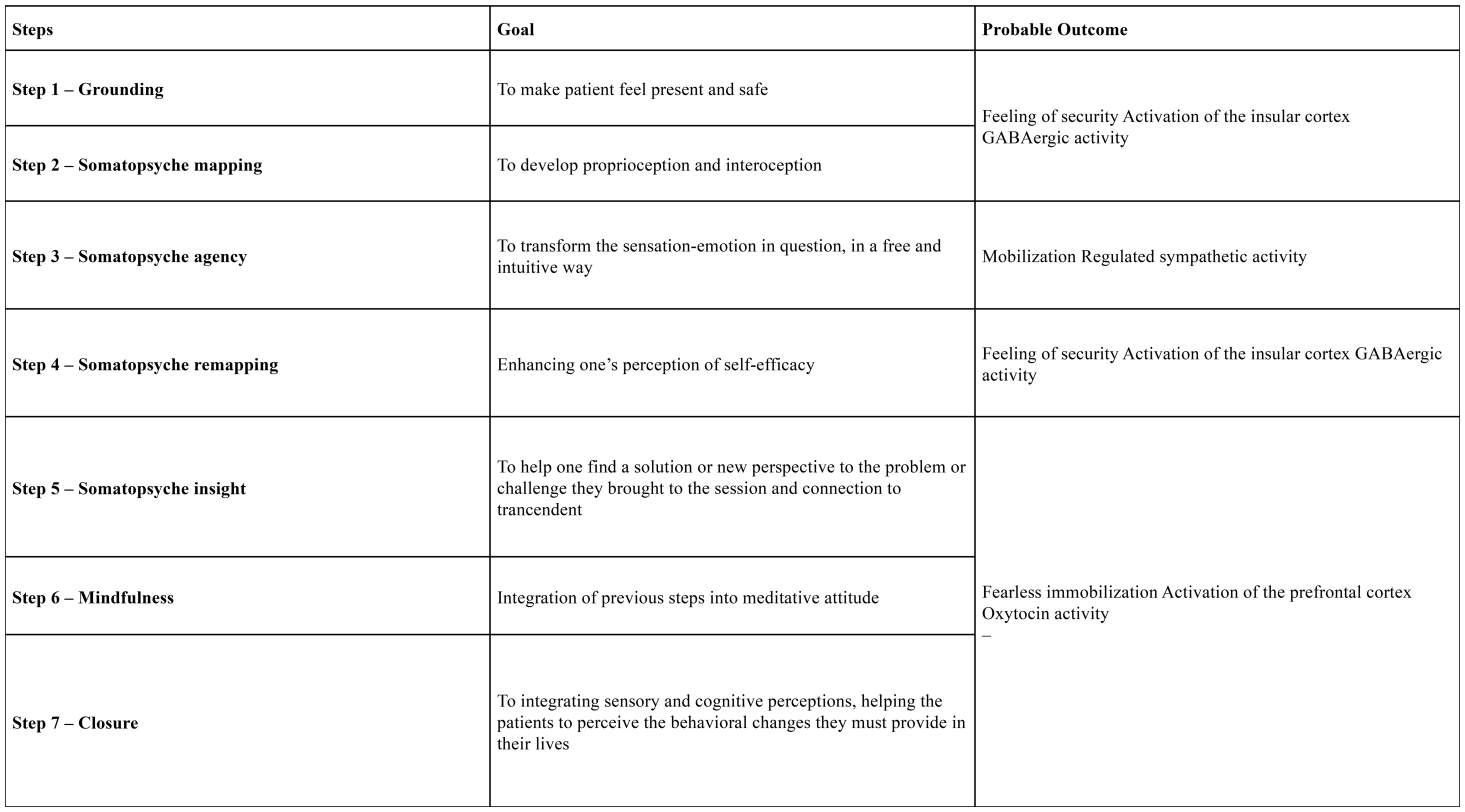
Diagram of the steps of ISP.

As a transdiagnostic intervention, SPI is built upon seven core steps (**Figure 3**), ideally implemented over approximately eight sessions, offering flexibility to adapt to individual patient needs. Its primary aim is to enhance emotional regulation, improve decision-making, and foster resilience, moving beyond mere symptom reduction towards promoting holistic well-being and human flourishing.

Crucially, the successful implementation of SPI is highly dependent on the patient’s clinical stability. While the intervention holds significant promise for a wide range of common mental complaints, including depression, anxiety, and substance use disorders, it is particularly suited for individuals in outpatient settings who are clinically stable. This focus is paramount because patients need to be in a stable condition to fully engage with and benefit from the protocol. The intervention leverages profound body-mind connection techniques, and in cases of acute psychotic or manic states, such intensive self-exploration could potentially disorganize rather than assist the patient. Therefore, applying SPI in such unstable conditions is not recommended, as it could lead to iatrogenic effects, hindering recovery instead of promoting it. SPI is thus best utilized with subacute or post-crisis patients experiencing emotional disconnection, as well as those presenting with less severe but persistent emotional dysregulation. For patients with specific comorbidities like severe emotional dysregulation, dissociative symptoms, or a history of trauma, adapted protocols with a gradual approach and more intensive follow-up are essential to ensure safety and maximize therapeutic outcomes.

A diagram illustrating the seven steps of the SPI is presented in **Figure 3**. For a comprehensive, session-by-session breakdown of the SPI protocol, including the detailed description of each practice and its theoretical basis, please refer to **Appendix**.

## 5 Discussion

This study primarily aimed to propose and substantiate the Somatopsyche Psychiatric Intervention (SPI), an innovative body-mind therapeutic approach designed to be integrated into psychiatric clinical practice. The main outcome of this work is the development of a structured protocol, comprising seven steps typically implemented over an eight-session framework. This intervention uniquely integrates concepts of body awareness, meditative practices, and contemporary neuroscience theories, presenting a significant potential for improving emotional regulation, decision-making, and resilience in psychiatric patients. SPI offers a holistic perspective on mental health treatment that extends beyond the traditional biomedical model, striving for well-being and human flourishing.

The SPI proposal is situated within the broader context of a growing convergence between body-mind practices and conventional psychiatry, a movement that has gained considerable momentum over the past two decades (3, 4). However, our approach distinguishes itself by offering a systematic and integrated methodology specifically tailored for the psychiatric context. While earlier studies, such as Goyal et al. (40), focused primarily on isolated mindfulness practices, SPI represents a substantial advancement by incorporating elements of bodily agency, somatopsyche mapping, and insight into a cohesive and integrated protocol. Furthermore, the introduction of the “Self-watcher” as a fifth level of embodiment provides a novel conceptual framework for understanding the advanced stages of body-self integration achieved through such interventions.

SPI differentiates itself from other structured body-focused approaches like Levine’s Somatic Experiencing (SE) (41) and Mindful Awareness in Body-Oriented Therapy (MABT) (42). While SE emphasizes interoception and includes steps analogous to Somatopsyche mapping (Step 2) and agency (Step 3), it typically does not feature specific steps dedicated to transcendent practice or formal mindfulness (Steps 5 and 6 in SPI). Similarly, MABT integrates interoceptive awareness for emotion regulation, sharing principles with SPI. However, SPI’s comprehensive protocol, which explicitly includes gratitude, celebration, and a distinct focus on advancing through explicit levels of embodiment culminating in the “Self-watcher,” provides a unique and expansive framework not explicitly found in these other models.

The theoretical framework of SPI is robustly supported by established scientific models. Its emphasis on interoception and proprioception aligns with recent findings by Seth and Critchley (43) on the relevance of interoception to emotional regulation and self-awareness. The incorporation of Porges’ Polyvagal Theory (2007) provides a solid neurophysiological foundation for understanding the dynamic interactions between bodily states and mental health, strengthening the interventions with robust scientific support. Moreover, SPI’s conceptualization is compatible with Damasio’s Somatic Marker Hypothesis (1994), which posits that bodily sensations inform decision-making, validating the intervention’s focus on the physical aspect of subjective consciousness (19). This theoretical-practical synthesis not only provides a basis for understanding the mechanisms through which SPI promotes therapeutic change but also contributes to the broader field of affective neuroscience.

The proposal to use SPI as a transdiagnostic approach aligns with current trends in psychiatry (12). This perspective is particularly relevant given the growing recognition of the limitations of traditional diagnostic classification systems and the increased interest in approaches addressing common underlying mechanisms across various mental disorders. The current global health context, marked by the somato-emotional impact of the COVID-19 pandemic (37), further underscores the need for such integrated approaches that can address exacerbated conditions like dissociation, alexithymia, and suicidality. SPI’s tailored protocols for specific comorbidities and neurodevelopmental conditions like Autism Spectrum Disorder (38, 39) highlight its adaptability and potential to provide targeted benefits for patients experiencing interoceptive dysfunction and altered embodiment.

A significant innovation within SPI is the inclusion of practices such as gratitude and celebration. These elements, often overlooked in traditional psychiatric interventions, have been shown to substantially promote well-being and reduce depressive and anxious symptoms (37). Integrating these positive psychology elements into SPI not only broadens its therapeutic potential but also aligns the intervention with a proactive stance on mental health promotion, moving beyond an exclusive focus on pathology towards human flourishing.

Despite its solid theoretical foundation and innovative integration of multiple body-mind approaches, it is imperative to acknowledge the limitations of this study. SPI, at this stage, remains primarily a theoretical model requiring rigorous empirical validation. Further research, particularly large-scale randomized clinical trials, is essential to establish its comparative effectiveness against conventional psychiatric approaches such as cognitive behavioral therapy (CBT) and pharmacotherapy. Furthermore, the applicability of SPI across diverse populations—including different age groups, cultural contexts, and varying levels of mental disorder severity—requires further investigation. While this narrative review aimed to substantiate SPI as a broad transdiagnostic psychiatric intervention supported by evidence-based theoretical models, it was not intended as an exhaustive review of all body-mind relationships and therapeutic practices.

Practical challenges for the implementation of SPI in clinical practice are also foreseeable. These include the need for adequate training of mental health professionals, its effective integration into existing healthcare services, and acceptance by both patients and practitioners accustomed to more traditional methodologies. However, as with other groundbreaking therapies, SPI must be assessed in real-world clinical populations through longitudinal studies aimed at establishing its immediate and long-term effects. Future research should also explore potential biological markers and process measures that can bridge the gap between body-mind-based interventions and symptom alleviation.

In conclusion, the Somatopsyche Psychiatric Intervention represents a significant advancement in the field, offering a holistic approach that emphasizes the inseparable unity of body and mind, in alignment with the latest World Health Organization guidelines for mental health care. By extending beyond mere symptom relief, SPI aims to foster happiness, well-being, and flourishing, promoting a more integrative vision of mental health. Nevertheless, it must be clearly acknowledged that SPI is primarily a theoretical model at this stage, and robust empirical validation through pilot studies and randomized controlled trials is essential to explore its practical applications and efficacy.

## Data Availability

The raw data supporting the conclusions of this article will be made available by the authors, without undue reservation.

## References

[1] Capitão CG Carvalho EB. Psychosomatics: Two approaches to the same problem. PSIC – J Psychol Vetor Publisher. (2006) 7:21–9.

[2] Ilyas MF Lukas GA Lado A Rahmayani SA Tan K Benedictus B. A bibliometric study of worldwide scientific literature on somatopsychics, (*1913–2022)* . Bratisl Med J/Bratisl Lek Listy. (2024) 125:68. doi: 10.4149/BLL_2024_68, PMID: 38943506

[3] Farb N Daubenmier J Price C Gard T Kerr C Barnaby D. Interoception, contemplative practice, and health. Front Psychol. (2015) 6. doi: 10.3389/fpsyg.2015.00763, PMID: 26106345 PMC4460802

[4] Farb N Daubenmier J Price CJ Gard T Kerr C Dunn BD. Body Awareness: a phenomenological inquiry into the common ground of body-mind therapies. Philos Ethics Humanit Med. (2011) 6:6. doi: 10.1186/1747-5341-6-6, PMID: 21473781 PMC3096919

[5] Tan Y Yan R Gao Y Zhang M Northoff M. Spatial-topographic nestedness of interoceptive regions within the networks of decision making and emotion regulation: Combining ALE meta-analysis and MACM analysis. Neuroimage. (2022) 260:119500. doi: 10.1016/j.neuroimage.2022.119500, PMID: 35872175

[6] Dunn BD Galton HC Morgan R Evans D Oliver C Meyer M. Listening to your heart. How interoception shapes emotion experience and intuitive decision making. Psychol Sci. (2010) 21:1835–44. doi: 10.1177/0956797610389191, PMID: 21106893

[7] Domschke K Stevens S Pfleiderer B Gerlach AL. Interoceptive sensitivity in anxiety and anxiety disorders: An overview and integration of neurobiological findings. Clin Psychol Rev. (2010) 30:1–11. doi: 10.1016/j.cpr.2009.08.008, PMID: 19751958

[8] Feeny NC Zoellner LA Fitzgibbons LA Foa EB. Exploring the roles of emotional numbing, depression, and dissociation in PTSD. J Trauma Stress. (2000) 13:489–98. doi: 10.1023/A:100778940933, PMID: 10948488

[9] Olsen VV Lugo RG Sütterlin S. The somatic marker theory in the context of addiction: contributions to understanding development and maintenance. Psychol Res Behav Manage. (2015) 8:187–200. doi: 10.2147/PRBM.S68695, PMID: 26185474 PMC4501162

[10] Paulus MP Stein MB. Interoception in anxiety and depression. Brain Struct Funct. (2010) 214:451–63. doi: 10.1007/s00429-010-0258-9, PMID: 20490545 PMC2886901

[11] Wiebking C de Greck M Duncan NW Tempelmann C Bajbouj M Northoff M. Interoception in insula subregions as a possible state marker for depression-an exploratory fMRI study investigating healthy, depressed and remitted participants. Front Behav Neurosci. (2015) 9:82. doi: 10.3389/fnbeh.2015.00082, PMID: 25914633 PMC4392695

[12] Barlow DH Farchione TJ eds. Applications of the Unified Protocol for Transdiagnostic Treatment of Emotional Disorders. New York, NY: Oxford University Press (2017).

[13] Sauer-Zavala S Gutner CA Farchione TJ Boettcher HT Bullis JR Barlow DH. Current definitions of “transdiagnostic” in development: a search for consensus. Behav Ther. (2017) 48:128–38. doi: 10.1016/j.beth.2016.09.004, PMID: 28077216

[14] Bohlmeijer E Westerhof G. The model for sustainable mental health: future directions for integrating positive psychology into mental health care. Behavior Therapy. (2021) 12:747999. doi: 10.3389/fpsyg.2021.747999, PMID: 34744925 PMC8566941

[15] Slade M. Measuring recovery in mental health services. Israel J Psychiatry and Related Sciences. (2010) 47(3):206–12. 21149985

[16] Freud S. The ego and the id, The Standard Edition of the Complete Psychological Works of Sigmund Freud: The Ego and the Id and Other Works (1923-1925). 19:1–66.

[17] Reich W. Character analysis (T. P. Wolfe, Trans.). Orgone Institute Press (1945). (Original work published 1933).

[18] Lowen A. Bioenergetics: Revolutionary therapy that uses the language of the body to heal the problems of the mind. Coward, McCann & Geoghegan (1975).

[19] Damasio AR. Design errors and the future of human life. Sci Am. (1994) 271:144. doi: 10.1038/scientificamerican1094-144, PMID: 7939563

[20] Damásio AR Grabowski TJ Bechara A Damásio H Ponto LL Parvizi J. Subcortical and cortical brain activity during the feeling of self-generated emotions. Nat Neurosci. (2000) 3:1049–56. doi: 10.1038/79871, PMID: 11017179

[21] Schmitt WA Brinkley CA Newman JP. Testing Damasio’s somatic marker hypothesis with psychopathic individuals: risk takers or risk averse? J Abnorm. Psychol. (1999) 108:538–43. doi: 10.1037//0021-843x.108.3.538, PMID: 10466278

[22] Suzuki A. Emotional functions of the insula. Brain Nerve. (2012) 64:1103–12. doi: 10.1038/79871, PMID: 23037601

[23] Wielgosz J Goldberg SB Kral TRA Dunne JD Davidson RJ. Mindfulness meditation and psychopathology. Annu Rev Clin Psychol. (2019) 15:285–316. doi: 10.1146/annurev-clinpsy-021815-093423, PMID: 30525995 PMC6597263

[24] Craig AD. How do you feel? Interoception: the sense of the physiological condition of the body. Nat Rev Neurosci. (2002) 3:655–66. doi: 10.1038/nrn894, PMID: 12154366

[25] Baer RA. Measuring mindfulness. Contemp Buddhism. (2011) 12:241–61. doi: 10.1080/14639947.2011.564842

[26] Ngô TL. Review of the effects of mindfulness meditation on mental and physical health and its mechanisms of action. Sante Ment Que. (2013) 38:19–34. doi: 10.7202/1023988, PMID: 24719001

[27] Luders E Cherbuin N Kurth F. Forever Young(er): potential age-defying effects of long-term meditation on gray matter atrophy. Front Psychol. (2015) 5:1551. doi: 10.3389/fpsyg.2014.01551, PMID: 25653628 PMC4300906

[28] Valluri J Gorton K Schmer C. Global meditation practices: A literature review. Holist Nurs Pract. (2024) 38:32–40. doi: 10.1097/HNP.0000000000000626, PMID: 37966989

[29] Goldberg SB Tucker RP Greene PA Davidson RJ Wampold BE Kearney DJ. Mindfulness-based interventions for psychiatric disorders: A systematic review and meta-analysis. Clin Psychol Rev. (2018) 59:52–60. doi: 10.1016/j.cpr.2017.10.011, PMID: 29126747 PMC5741505

[30] Eberth J Sedlmeier P. The effects of mindfulness meditation: A meta-analysis. Mindfulness. (2012) 3:174–89. doi: 10.1007/s12671-012-0101-x

[31] Barlattani T Grandinetti P Cintio AD Ferri G Di Gregorio V Iannitelli A. Glymphatic system and psychiatric disorders: A rapid comprehensive scoping review. Curr Neuropharmacol. (2024) 22:2016–33. doi: 10.2174/1570159X22666240130091235, PMID: 39234773 PMC11333792

[32] Hudak PL McKeever P Wright JG. Unstable embodiments: a phenomenological interpretation of patient satisfaction with treatment outcome. J Med Humanit. (2007) 28:31–44. doi: 10.1007/s10912-006-9027-4, PMID: 17333378

[33] Garland EL Hanley AW Baker AK Howard MO. Biobehavioral mechanisms of mindfulness-based interventions. Neurosci Biobehav Rev. (2022) 137:104645. doi: 10.1007/s12671-012-0101-x 35367513

[34] Porges SW. The polyvagal perspective. Biol Psychol. (2007) 74(2):116–43. doi: 10.1016/j.biopsycho.2006.06.009, PMID: 17049418 PMC1868418

[35] Hughlings Jackson J. On the anatomical and physiological localization of movements in the brain. In: Taylor J, editor. Select writings of John Hughlings Jackson Vol i, vol. 52 . Basic Books, New York (1958).

[36] Dana D. The Polyvagal Theory in Therapy: Engaging the Rhythm of Regulation. WW Norton & Company (2018).

[37] Komase Y Watanabe K Hori D Nozawa K Hidaka Y Iida M. Effects of gratitude intervention on mental health and well-being among workers: A systematic review. J Occup Health. (2021) 63:e12290. doi: 10.1002/1348-9585.12290, PMID: 34762326 PMC8582291

[38] Barlattani T D’Amelio C Capelli F Cianfarani L Manna F Orsini B. Suicide and COVID-19: a rapid scoping review. Ann Gen Psychiatry. (2023) 22:10. doi: 10.1186/s12991-023-00441-6, PMID: 36932453 PMC10020759

[39] Barlattani T D’Amelio C Cavatassi A Sani G Koukopoulos A. Autism spectrum disorders and psychiatric comorbidities: a narrative review. J Psychopathol. (2023) 29:3–24.

[40] Goyal M Singh S Sibinga EM Gould NF Rowland-Seymour A Sharma R. Meditation programs for psychological stress and well-being: a systematic review and meta-analysis. JAMA Intern Med. (2014) 174:357–68. doi: 10.1001/jamainternmed.2013.13018, PMID: 24395196 PMC4142584

[41] Payne P Levine PA Crane-Godreau MA. Somatic experiencing: using interoception and proprioception as core elements of trauma therapy. Front Psychol. (2015) 6:93. doi: 10.3389/fpsyg.2015.00093, PMID: 25699005 PMC4316402

[42] Price CJ Hooven C. Interoceptive awareness skills for emotion regulation: theory and approach of mindful awareness in body-oriented therapy (MABT). Front Psychol. (2018) 9:798/full. doi: 10.3389/fpsyg.2018.00798/full 29892247 PMC5985305

[43] Seth AK Critchley HD. Extending predictive processing to the body: emotion as interoceptive inference. Behav Brain Sci. (2013) 36(3):227–8. doi: 10.1017/S0140525X12002270, PMID: 23663284

