# Supplementary material for: Theory and conception of Somatopsyche Psychiatric Intervention: a transdiagnostic body-mind intervention for psychiatric practice
Source: Front Psychiatry. 2025 Aug 19;16:1644739. doi: 10.3389/fpsyt.2025.1644739 (PMC12401917; doi:10.3389/fpsyt.2025.1644739)
Supplement: Supplementary file 1 [file SupplementaryFile1.docx]

**Appendix A: Detailed Somatopsyche Intervention (SPI) Protocol**

This appendix provides a comprehensive, session-by-session description of the Somatopsyche Psychiatric Intervention (SPI) protocol, outlining the sequence of practices and their underlying theoretical foundations. The SPI is structured in seven core steps, developed over approximately eight sessions, though flexibility is maintained to add or subtract one or two sessions based on individual or group needs. The intervention can be performed both in groups and individually. In group settings, a psychiatric assessment conducted individually must precede the intervention to establish the doctor-patient relationship and identify the patient's primary complaint or issue.

Each session begins with a brief psychoeducational introduction about the fundamentals and goals of the exercises to be performed. Importantly, steps practiced in previous sessions are revisited and reinforced, with new steps introduced progressively. The seven core steps are fully introduced by the 4th session. From the 5th to the 8th session, the complete set of seven steps is reproduced at the opening of each session. As the treatment advances, the psychoeducational content is gradually reduced to allow more space for practical application.

Every session ideally begins with the therapist inquiring about the patient's experiences from the preceding week. Each proposed exercise is preceded by an explanation of its purpose, and upon completion, patients are encouraged to share their perceptions and feelings. The SPI is designed to be delivered in a trusting, safe, and cooperative context, necessitating a nonjudgmental and embracing attitude from the clinician.

**Session 1**

**Grounding and Embodiment: Connecting with Your Body and the Present Moment**

Upon the patient's initial visit, the clinician first ascertains the demand that precipitated help-seeking through an anamnesis. Following this, the clinician introduces the theory of the body-mind relation, the SPI itself, and the fundamental concepts of body awareness and levels of embodiment. During this theoretical introduction, the clinician presents the five levels of embodiment (as described in Section 2 of the main text) and invites patients to identify which level best reflects their current status. After this theoretical introduction, the practical component commences, aiming to shift the patient’s perception toward their own body and connection with the present moment ("here and now"). To achieve this, the therapist introduces Step 1.

- **Step 1: Grounding (5 min)**: The primary objective of this step is to help the patient feel safe and present. To provide a secure environment, it is essential for the clinician to adopt an empathetic attitude, maintain eye contact, and use a friendly voice intonation and facial expression. Relaxing background music is also incorporated and maintained throughout the seven steps. To facilitate a deeper connection with themselves and the present moment, the clinician proposes the grounding exercise sequence (Figure 1), in which the individual stands with hips slightly medially rotated, knees slightly flexed, and the torso flexed forward and downward. In this posture, a natural vibration may occur, typically starting in the legs and extending throughout the entire body (Lowen, 1975)^18^. This exercise enhances body awareness and the feeling of presence. Positioned in front of the patient, the clinician guides the patient to adopt the described posture, encourages free breathing, asks them to rest their chin on their chest, and allow the torso to gently and progressively incline forward while their legs and feet sustain the position. It is clarified that if any emotion arises during the exercise, it should be allowed to express without judgment. The sense of security facilitated by grounding is fundamental for the individual to maintain a focus of consciousness on the present experience, fostering trust and enjoyment in the connection with the present moment, which is central to contemplative well-being.

**Figure 1: Grounding exercise sequence**


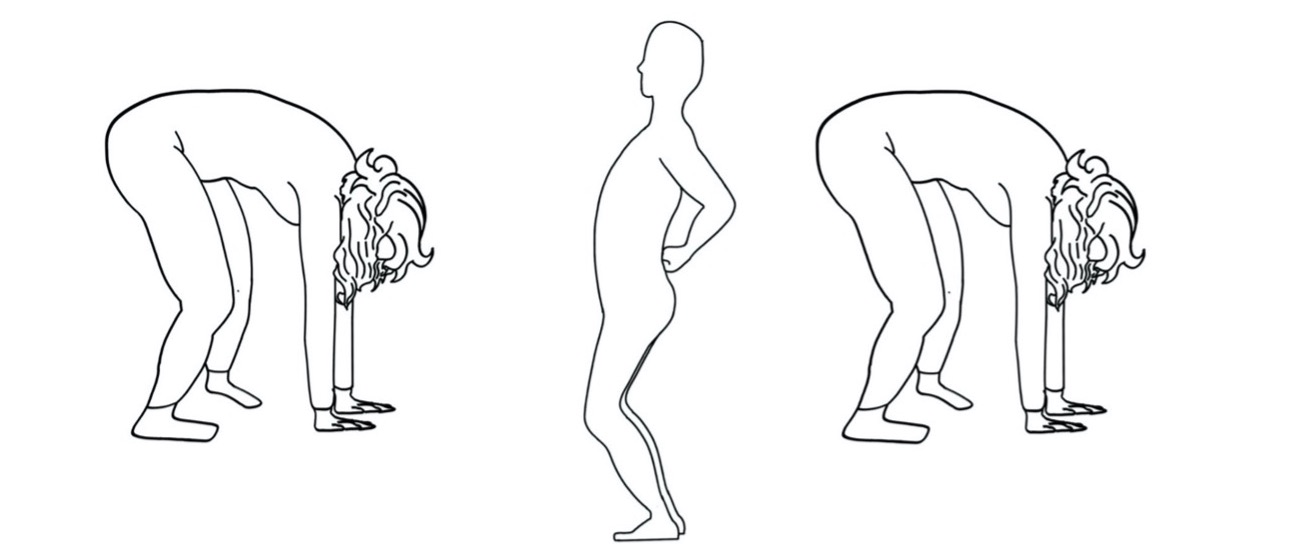


**Session 2**

**Mapping Sensations: The Mind Within the Body**

In the second session, the clinician introduces the following key concepts:

1. **The principle of mapping body sensations**: This concept highlights those specific emotions—such as anger, anxiety, and happiness—are often preceded by physical sensations, which precede the conscious awareness of these emotions.
2. **Muscular tensions**: Chronic muscular tensions are often linked to emotions that have not been properly processed or elaborated. As patients develop a deeper perception of muscular tension, they may also become aware of underlying emotions like anger, sadness, and/or fear, which are commonly repressed.
3. **Unconditional self-acceptance**: The therapist introduces this concept and invites each participant to adopt an attitude of complete self-acceptance, viewing themselves as valuable human beings worthy of affection, independent of demonstrations of self-efficacy or the approval and love of others. The goal is to support patients in engaging with their emotions in a self-compassionate manner.

Following this initial exchange, the practical part of the session begins. The therapist repeats the grounding exercise (Step 1) and then introduces the first component of Step 2, focusing on a more detailed observation of bodily sensations.

- **Step 2 (first practice): Superficial Somatopsyche Mapping (2 min)**: The main goal of this step is to develop proprioception and interoception. After performing Step 1 (grounding), the clinician guides patients to stand with eyes closed, directing their attention to bodily sensations, starting from the soles of the feet and moving upwards to the top of the head. The attention then slowly descends back to the soles of the feet. It is reinforced that this observation must be performed with a nonjudgmental and accepting mindset, welcoming all perceptions and emotions that may emerge into consciousness. If patients identify any points of tension, they are encouraged to move their body slowly to achieve relaxation. Connecting with body signals, particularly interoceptive signals, has been associated with improved emotion regulation and decision-making, thereby facilitating cognitive reframing and preparing the individual for nonreactive responses.

**Session 3**

**Somatopsyche Agency: Transforming Sensations with Intuition and Creativity**

The third session is dedicated to introducing two pivotal concepts:

1. **Deep Somatopsyche Mapping**: The clinician guides patients in a detailed characterization of physical sensations correlated with specific aspects they wish to modify. For instance, if a patient wishes to address fear of public speaking, the clinician guides them to recall a vivid scene evoking this fear. A brief pause follows, allowing the patient to expand their consciousness of the connection between body sensations and this specific emotion.
2. **Agency**: The concept of agency and its relevance to promoting well-being is introduced.

After this conceptual introduction, the practical component of the session begins. Steps 1 (grounding) and 2 (somatopsyche mapping) are repeated, followed by the introduction of Step 3.

- **Step 2 (second exercise): Deep Somatopsyche Mapping (8 min)**: The clinician prompts patients to identify the body parts where they perceive the specific aspects they wish to address. They are then encouraged to characterize these perceptions in detail: inquiring about texture, temperature, color, shape, contour, size, and any associated sounds. If patients perceive sounds, the therapist may guide them to reproduce these sounds.
- **Step 3: Somatopsyche Agency (10 min)**: After the detailed characterization of bodily discomfort (Step 2), patients are encouraged to find movements, body positions, and/or sounds that intuitively and creatively help them transform the sensation-emotion in question. It is strongly recommended that they do not remain still, as movement plays a fundamental role in Somatopsyche agency. This step aims to use body movements, guided by intuition and creativity, to transform unpleasant sensations. It serves as a tool to fortify self-responsibility and a sense of self-efficacy, thereby promoting well-being.

**Session 4**

**Insight and Transcendence: Discovering Inner Wisdom and New Perspectives**

The fourth session introduces the concepts of insight and transcendence. An interactive moment allows participants to verbalize their understanding of each concept and how they navigate them in daily life. Following this, the clinician leads the execution of Steps 1 through 3 and then introduces Steps 4 and 5.

- **Step 4: Superficial and Deep Somatopsyche Remapping (5 min)**: Returning to the perception initially mapped within this session, the clinician guides the patients' attention to observe what has changed in terms of body sensations from the beginning of the session to the present moment (e.g., sensation-emotion quality, temperature, texture, form, color, and sound). For example, an initial perception in the stomach described as "red with an acute sound" might transform into "blue with a grave and more pleasant sound." The execution of a new mapping after the Somatopsyche agency exercise aims to enhance the patient's perception of self-efficacy by allowing them to compare how they felt before and after actively engaging with their sensations.
- **Step 5: Somatopsyche Insight (5 min)**: The clinician guides patients to close their eyes and place both hands over the center of their chest to encourage openness and foster a connection with transcendence according to their personal understanding (e.g., God, the universe, enlightenment, knowledge, or internal wisdom for agnostics and atheists). Patients are then directed to pay attention to any word, phrase, or image that may arise in their consciousness, which is intended to help them with the problem or challenge they wish to address in the session. This process allows them to access their inner wisdom and find solutions to their difficulties.

**Session 5**

**Meditation Keys: Cultivating Calmness and Mental Clarity**

The fifth session is dedicated to introducing the concepts of the "three keys of meditation" and mindfulness, along with their associated global health benefits. These three keys are essential guidelines that facilitate meditative practice, fostering a more effective and enriching experience:

1. **Relaxation of the body and mind**: To ensure a state of calmness and ease in both physical and mental realms.
2. **Observation**: To observe everything that arises in consciousness, maintaining awareness of thoughts and sensations without becoming attached to them.
3. **Non-judgment**: To refrain from judging what is observed or experienced, allowing feelings to unfold without labeling them as good or bad.

After this theoretical introduction, the practical part begins with the execution of Steps 1, 2, 3, 4, and 5, followed by the introduction of Step 6, which comprises the experience of mindfulness, and Step 7, which provides closure.

- **Step 6: Mindfulness (5 min)**: The clinician guides the patient to sit comfortably, ease their breathing, and maintain a perceptive state with body-mind relaxation, observing internal and external stimuli in an atmosphere of acceptance and non-judgment.
- **Step 7: Closure (5 min)**: In this final step of the session, with the clinician's assistance, patients are invited to seek correlations between insights elicited during the session, their personal biographies, and their life context. This step is critically important for integrating sensory and cognitive perceptions, helping patients recognize the behavioral changes they need to implement in their lives.

**Session 6**

**Discernment and Regulation: Identifying Patterns and Bodily Responses**

With all seven steps now introduced and practiced, Session 6 focuses on developing the patients' resourcefulness. This involves fostering their ability to discriminate between adaptive responses (thoughts, actions, and choices that promote well-being) and maladaptive ones (those that induce discomfort, fatigue, and reduce body vitality). Patients are guided to connect with a thought, action, or choice that currently challenges them. When commencing the routine of steps, during the deep Somatopsyche mapping (Step 2), the patient is oriented to locate where in their body the sensation provoked by this challenging thought, action, or choice arises. This establishes a direct connection between life challenges, body responses, insight, and decision-making. Additionally, the therapist introduces body vibration as a tool to address mental stress. This technique, commonly employed in active meditation practices, aids in relaxation and the alleviation of muscular tension, thereby enhancing patients' agency over their emotions. Patients are instructed to stand with their feet hip-width apart, knees slightly bent, and to produce a gentle shake with their entire body. They then gradually increase the intensity of the vibration, directing their attention to the physical sensation stemming from the soles of their feet and rising through the legs to the whole body^32^ . This specific exercise is introduced during Step 3 (Somatopsyche Agency) to enhance the transformational aspect of movement.

**Session 7**

**The Last Call: The Best Time for Change**

The seventh session marks the opening of the final phase of treatment, inviting patients to assess the outcomes of the intervention up to this point. Before performing the seven steps, patients are prompted to evaluate and share what they have achieved and what remains between them and their original goals when they began treatment. The seven steps, particularly body mapping (Step 2), will then be performed with the specific goal of locating in one’s own body the sensation associated with whatever remaining challenges or objectives the patients wish to address. The agency exercise (Step 3) and subsequent steps are then performed to facilitate these final necessary changes. The mindset of this specific session is conceptualized as a "last call" for subjective and behavioral changes that patients may still need to implement, and this should be expressly communicated by the clinician.

**Session 8**

**Gratitude and Celebration: Lifelong Keys to Well-being**

The eighth session is designed to integrate the concepts of gratitude and celebration for everything experienced and achieved throughout the process, reflecting on how patients perceive themselves now. In a more colloquial analogy, the therapist can refer to this intervention as activating a "mind GPS," meaning that by mentally reviewing their therapeutic journey to identify achievements and new learnings, they can pinpoint their current position in life. Finally, the celebration component reinforces feelings of gratitude and self-congratulation for the effort made and the results obtained, even if they do not precisely match initial expectations. After this theoretical introduction and exchange, the therapist proposes a new instruction: inviting patients to recall memories of their experiences, insights, and changes from the beginning of the process until the present moment, choosing the most significant recollection. With this memory in mind, the patient then initiates the 7-step process one last time.

At this point, the clinician reintroduces the scale of embodiment used in the first session, requesting that patients perform a reassessment of their body status. In Step 2 (somatopsyche mapping), the patient should endeavor to locate within their body the sensations evoked by this specific memory. In Step 3 (agency), the clinician encourages individuals to first experience gratitude and then celebrate, often inviting them to engage in dance. In group interventions, for more inhibited patients, the therapist might offer a blindfold to facilitate engagement in dancing. In Step 4 (second somatopsyche mapping), the individual is invited to perceive any possible changes that occurred after practicing the gratitude and celebration exercise. The other steps (5, 6, and 7) then proceed as in previous sessions. In this concluding session, the therapist encourages the patient to continue practicing the seven steps taught during SPI whenever an unpleasant emotional state or challenging situation emerges in their lives, thus framing the protocol as a self-care tool and a strategy for ongoing health promotion.
